# Preoperative Radiation Boost Results in Low Rates of Re-excision and Reduced Locoregional Treatment Time in Breast Cancer Patients

**DOI:** 10.1245/s10434-025-18089-9

**Published:** 2025-08-20

**Authors:** Molly A. Chakraborty, Zohaib K. Sherwani, Lakshmi Rekha Narra, Zeinab Abou Yehia, Nisha Ohri, Taoran Cui, Firas Eladoumikdachi, Maria J. Kowzun, Shicha Kumar, Lindsay Potdevin, Deborah L. Toppmeyer, Bruce G. Haffty

**Affiliations:** 1 Rutgers New Jersey Medical School, Newark, NJ USA; 2 Department of Radiation Oncology, Rutgers Cancer Institute of New Jersey, New Brunswick, NJ USA; 3 Division of Surgical Oncology, Rutgers Cancer Institute of New Jersey, New Brunswick, NJ USA; 4 Division of Medical Oncology, Rutgers Cancer Institute of New Jersey, New Brunswick, NJ USA

**Keywords:** Preoperative boost, Radiation boost, Breast cancer, Re-excision, Whole breast radiotherapy, Breast conserving surgery, Lumpectomy, Locoregional treatment time

## Abstract

**Background:**

We conducted a prospective, phase II trial in which a radiation tumor bed boost was delivered preoperatively instead of the standard postoperative approach for breast cancer patients. We hypothesized that a preoperative boost would result in a lower rate of re-excision and a shorter duration of locoregional therapy compared with a postoperative boost.

**Methods:**

Patients in this trial (NCT04871516) received a boost of 13.32 Gy in 4 fractions, followed by lumpectomy and whole-breast radiotherapy of 36.63 Gy in 11 fractions. The re-excision rate in this trial was compared with the literature-reported rate and with a cohort of contemporary patients at our institution. The time from initial diagnostic biopsy to completion of radiotherapy was compared with the same contemporary cohort and with patients treated in a previous prospective trial at our institution (NCT00909909) with the same fractionation scheme.

**Results:**

Among 89 patients who were included, three (3.4%) required re-excision for inadequate surgical margins. This rate was lower than the literature-reported rate of 17.2% (*p* = 0.0005) and was also lower than the contemporary cohort (13.48%, *p* = 0.015). The median locoregional treatment time was 109 days (42–258) in this trial, which was shorter than the median treatment time in both the previous trial [122 days (62–311), *p* = 0.0002] and the contemporary cohort [126 days (74–278), *p* ≤ 0.0001].

**Conclusions:**

Lower re-excision rates and shorter time from initial diagnosis to completion of radiotherapy, as observed in this phase II trial, may reduce treatment costs and risk of wound complications, improve treatment experience, and enable earlier initiation of systemic therapy.

Early-stage breast cancer and ductal carcinoma in situ (DCIS) are commonly treated with breast-conserving surgery (BCS) followed by whole-breast radiotherapy (WBRT). This is then followed by a radiation tumor bed boost, depending on patient age and disease characteristics.^1^ Given that most local recurrences occur within or near the original tumor bed, studies have demonstrated that the addition of a tumor bed boost after WBRT can reduce the local recurrence risk, with the highest absolute benefit observed in younger patients.^2–6^

A radiation tumor bed boost is delivered after BCS and WBRT by convention and due to limited historic data demonstrating significant toxicities and poor wound healing after preoperative breast radiotherapy.^7,8^ However, these studies primarily focused on WBRT, in contrast to tumor bed radiotherapy alone.

A lower preoperative dose, such as the dose used for a boost, may allow for lesser toxicity and may also offer the following advantages: more accurate tumor volume delineation, reduced normal breast tissue irradiation, delivery of radiation in a non-hypoxic environment, and earlier stimulation of an immune response. Furthermore, it may allow for downsizing of the tumor prior to BCS, which may result in lower re-excision rates following initial lumpectomy.^9^

An estimated 16–19% of women currently undergo re-excision after initial lumpectomy.^10–12^ This significantly high percentage of patients needing re-excision is concerning because additional surgery results in higher treatment costs, increases the risk of operative/wound complications, and is distressing to patients. It also delays the initiation of further local and systemic therapy.^11^ A preoperative radiation boost may additionally allow for a reduced locoregional treatment time, as patients can generally start radiotherapy in a shorter time frame in comparison with undergoing BCS due to operating room scheduling limitations and other logistics.

We recently conducted a phase II clinical trial in which the radiation boost was delivered preoperatively rather than postoperatively, otherwise utilizing the same radiation fractionation scheme established by our institutional SHoRT-B trial.^13^ The initial results of the primary endpoint, the incidence of wound complications, was reported to be acceptable by Yehia et al.^9^ We hypothesized that the treatment regimen in this trial may result in a lower re-excision rate following initial lumpectomy, and reduce the time interval from initial diagnosis to completion of locoregional therapy.

## Methods and Materials

### Trial Protocol

This was a single-arm, prospective, phase II clinical trial (NCT04871516). The Institutional Review Board (IRB) at Rutgers University approved this protocol, and all patients provided written informed consent prior to enrollment in the trial. The details of the protocol and initial results have been previously reported.^9^ In brief, patients with mammogram-detected, biopsy confirmed, clinically node-negative, non-metastatic, early-stage invasive breast cancer or DCIS (cTis-2, cN0, cM0) who were eligible for BCS were enrolled in the trial. Patients then obtained a breast magnetic resonance imaging (MRI) scan that was fused to the treatment planning computed tomography (CT) scan and mammographic data for target delineation. This study included all patients who were actively enrolled in the trial and completed radiotherapy at the time of data acquisition (patients who underwent biopsy between June 2021 and October 2023).

All patients were discussed at our institutional multidisciplinary tumor board prior to initiation of treatment. Patients were simulated in either the supine or prone position on a breast board per the treating radiation oncologist’s discretion. The gross tumor volume (GTV) was outlined using the clip placed at the time of biopsy as the epicenter, utilizing the MRI fusion and mammographic data for accurate delineation. A dose of 13.32 Gy over 4 daily fractions was prescribed to the boost planning target volume using either photons or electrons. Patients then underwent lumpectomy with or without sentinel lymph node biopsy 1–3 weeks after radiation. If a patient required re-excision after initial lumpectomy, this was also performed prior to WBRT. After 3–8 weeks of recovery from surgery, patients had repeat CT simulation for the whole-breast phase of radiation. A dose of 36.63 Gy over 11 daily fractions was prescribed to the whole breast. Pathologically node-positive patients were allowed to be treated on the trial inclusive of internal mammary nodes, at the discretion of the treating radiation oncologist. This institutional fractionation scheme has previously been used in trials over the past 15 years in both lumpectomy and post-mastectomy patients.^13,14^ If a patient requires adjuvant chemotherapy, this is given after the completion of WBRT. Patients in this trial are serially followed for 3 years, during which toxicity, and clinical and cosmetic outcomes will be collected. Clinical trial records and patient charts were reviewed to obtain patient demographics, disease characteristics, and treatment details for this study.

### Comparison Patient Groups

Two other groups of early-stage breast cancer patients treated at our institution were utilized to draw comparisons between the re-excision rate and treatment times observed in the current trial. Comparison of re-excision rates was done with the literature-based rate as well as with a contemporary cohort of patients treated at our institution with the more conventional lumpectomy and postoperative WBRT and boost. This group of patients was treated with the Canadian hypofractionation regimen (postoperative WBRT of 2.67 Gy × 16 fractions with or without a boost of 2.5 Gy × 4 fractions).^15^ All patients in this group were biopsied between June 2021 and October 2023 to match the biopsy time frame of patients in the preoperative boost trial (*n* = 89). Patients receiving chemotherapy prior to radiotherapy were excluded, but patients were otherwise consecutive. It should be noted that many patients in this trial may have been eligible for the preoperative boost trial, but it is difficult to determine how many may have been approached about participation and how many refused. In this cohort, both the re-excision rates and treatment times were compared.

The second group of patients were from the SHoRT-B clinical trial, with the protocol details previously described.^13^ In brief, these patients received the same fractionation scheme as the current trial, but with a postoperative radiation boost (WBRT of 36.63 Gy over 11 fractions, followed by a boost of 13.32 Gy over 4 fractions). These patients were biopsied between February 2015 and December 2019 (*n* = 48). In this group of patients, we compared the treatment times only and not the re-excision rates, since these patients were not contemporary. Patient records were reviewed retrospectively to obtain demographics, disease characteristics, and treatment details for both groups, in accordance with the IRB protocols.

### Definition of Endpoints

Re-excision: Patients requiring at least one re-excision after BCS (lumpectomy), or a patient who was recommended to receive a re-excision but declined.

Treatment time: Defined as the time from initial diagnostic biopsy to completion of adjuvant radiotherapy.

### Statistical Analysis

A 95% confidence interval (CI) of the re-excision rate in the current trial was calculated using the Wilson Score Interval. The Chi-square goodness-of-fit test was used to compare the re-excision rate to 17.2%, as this was the re-excision rate found in a large national study of Medicare claims that only analyzed patients after the 2014 ‘no tumor on ink’ guideline, as we wanted to draw a comparison with national trends.^11^ A Chi-square test of independence was used to compare the re-excision rate in the current clinical trial with the re-excision rate of the contemporary patients treated with the Canadian hypofractionation regimen in order to draw an institutional comparison. Two-sample t-tests for quantitative data and a Chi-square test of independence/Fisher’s exact tests for categorical data were used to compare relevant characteristics between patient groups. Logistic regression was used to assess the relationship between any variable found to be significantly different between groups and re-excision status.

The treatment time for patients in the current trial was compared with that of patients in the SHoRT-B trial using a one-tail Welch’s t-test, and compared with the treatment time of the contemporary Canadian hypofractionation patients using a one-tail two-sample t-test.

## Results

### Demographic and Disease Characteristics

A total of 89 patients were included in the current trial, with a median age of 63 years (range 40–79). Most patients [74 (83.1%)] had invasive disease and 15 (16.9%) patients had DCIS. The median pathologic tumor size was 10 mm, and 4 patients (4.5%) had node-positive disease. Most patients, 82 (92.1%) had estrogen receptor (ER)-positive disease, 74 (83.1%) patients had progesterone receptor (PR)-positive disease, and 85 (95.5%) patients had human epidermal growth factor receptor 2 (HER2)-negative disease. On histology, 21 (23.6%), 51 (57.3%), and 11 (12.4%) patients had grade 1, 2, and 3 disease, respectively. The demographic and disease characteristics were relatively well-balanced between the preoperative boost and comparison groups, as summarized in Table 1.

**Table 1:** Demographic and disease characteristics

	Preoperative boost [*n* = 89]	Postoperative boost [*n* = 48]	Contemporary Canadian hypofractionation patients [*n* = 89]
Dose fractionation	3.33 Gy × 4 fx boost → surgery → 3.33 Gy × 11 fx WBRT	Surgery → 3.33 Gy × 11 fx WBRT → 3.33 Gy × 4 fx boost	Surgery → 2.67 Gy × 16 fx → 2.50 Gy × 4 fx boost
Age, years [median (range)]	63 (40–79)	58 (35–80)	61 (40–84); *p* = 0.06
Race			
White	63 (70.8)	32 (66.7)	30 (33.7)
Black	14 (15.7)	8 (16.7)	5 (5.6)
Asian	6 (6.7)	6 (12.5)	30 (33.7)
Other	2 (2.2)	1 (2.1)	23 (25.8) *p* < 0.001^a^
Ethnicity			
Non-Hispanic	72 (80.9)	42 (87.5)	74 (83.1)
Hispanic	17 (19.1)	5 (10.4)	15 (16.9) *p* = 0.70
Histology			
DCIS	15 (16.9)	12 (25.0)	35 (39.3)
IDC	59 (66.3)	30 (62.5)	48 (53.9)
ILC	12 (13.5)	4 (8.3)	2 (2.2)
Other invasive	3 (3.4)	0 (0.0)	4 (4.5) *p* = 0.002^a^
pT, mm [median (range)]	10 (0–37)	11 (1–49)	11.5 (0–72) *p* = 0.12
Node status			
N0	85 (95.5)	45 (93.8)	87 (97.8)
N+	4 (4.5)	2 (4.2)	2 (2.2) *p* = 0.68
Laterality			
Right	49 (55.0)	28 (58.3)	48 (53.9)
Left	40 (44.9)	17 (35.4)	41 (46.1) *p* = 0.88
ER status			
ER+	82 (92.1)	36 (75.0)	85 (95.5)
ER−	7 (7.9)	10 (20.8)	4 (4.5) *p* = 0.54
PR status			
PR+	74 (83.1)	28 (58.3)	79 (88.8)
PR−	15 (16.9)	18 (37.5)	9 (10.1) *p* = 0.27
HER2 status			
HER2+	4 (4.5)	8 (16.7)	1 (1.1)
HER2−/NR	85 (95.5)	38 (79.2)	88 (98.9) *p* = 0.37
Grade			
1	21 (23.6)	1 (2.1)	13 (14.6)
2	51 (57.3)	27 (56.3)	54 (60.7)
3	11 (12.4)	17 (35.4)	20 (22.5) *p* = 0.11

Data are expressed as *n* (%) unless otherwise specified

*Gy* Gray, *fx* fractions, *WBRT* whole-breast radiation therapy, *DCIS* ductal carcinoma in situ, *IDC* invasive ductal carcinoma, *ILC* invasive lobular carcinoma, *pT* pathologic tumor size, *ER* estrogen receptor, *PR* progesterone receptor, *HER2* human epidermal growth factor receptor 2, *NR* no record

^a^No association was found with re-excision status using logistic regression

### Re-excision Rate

Three patients (3.4%, 95% CI 1.15–9.45%) in the preoperative boost trial underwent re-excision after BCS for inadequate surgical margins, which is significantly less than the literature-reported rate of 17.2% (*p* = 0.0005), and was also significant when compared with the re-excision rate of the Canadian hypofractionation cohort (13.48%; *p* = 0.015). Re-excision and surgical margin data for the two treatment groups are summarized in Table 2. For the three patients requiring re-excision in the preoperative boost trial, all three had cavity shave margins performed during their surgery. One patient had a pT1c invasive ductal carcinoma (IDC), one patient had a pT2 ILC, and the remaining patient had a 2.5 mm DCIS lesion. Of the characteristics in Table 1, only race and histology were found to be significantly different between groups (*p* < 0.001 and *p* = 0.002, respectively). No relationship was found between these two variables and re-excision status (*p* = 0.84 and *p* = 0.09, respectively).

**Table 2:** Re-excision, surgical margin, and oncoplastic surgery status

	Preoperative boost [*n* = 89]	Contemporary Canadian hypofractionation patients [*n* = 89]
Initial margin status		
Positive	6 (6.7)	10 (11.2)
Close	19 (21.3)	22 (24.7)
Negative	64 (71.9)	57 (64.0)
Final margin status		
Positive	4 (4.5)	2 (2.2)
Close	18 (20.2)	20 (22.5)
Negative	67 (75.3)	67 (75.3)
Re-excision		
Yes	3 (3.4)	12 (13.5)
No	86 (96.6)	77 (86.5)
Oncoplastic		
Yes	24 (27.0)	25 (28.1)
No	65 (73.0)	64 (71.9)
Cavity shave margins		
Yes	42 (47.2)	67 (75.3)
No	45 (50.6)	22 (24.7)
No record	2 (2.2)	
Intraoperative radiography		
Yes	81 (91.0)	88 (98.9)
No	6 (6.7)	1 (1.1)
No record	2 (2.2)	

Data are expressed as *n* (%)

Initial margin margin status after initial lumpectomy, *final margin* margin status at the time of radiotherapy (after any additional re-excisions if applicable), *close* ≤ 2 mm

### Treatment Time

The time from initial diagnosis to completion of adjuvant radiotherapy in the preoperative boost trial was 109 days (range 42–258). This was significantly lower than the treatment time for the postoperative boost trial patients (median 122 days, range 62–311, *p* = 0.0002), and for the contemporary Canadian hypofractionation patients (median 126 days, range 74–278, *p* < 0.0001). One patient in the Canadian hypofractionation group, who chose to delay radiation therapy for personal reasons, was not included in this analysis due to her treatment time being a significant outlier. For the three patients in the preoperative boost trial who underwent re-excision, the median locoregional treatment time was 130 days, in comparison with a median of 109 days for the entire group. In the Canadian hypofractionation group, the median locoregional treatment time was 134 days for patients undergoing re-excision, in comparison with 126 days for the entire group.

## Discussion

In this study, we have first demonstrated a lower re-excision rate following BCS with a preoperative radiation boost. A preoperative radiation boost may allow for decreased tumor size at the time of BCS, which correlates with a decreased risk of positive margins and thus decreased need for re-excision.^16^ Lower re-excision rates can lead to reduction in treatment costs, improve patient experience, improve cosmetic results, and may reduce surgical complications allowing for the earlier initiation of additional therapies.^11^

Notably, re-excision rates may differ between institutions and individual surgeons due to varying uses of techniques that have been shown to decrease re-excision rates, such as cavity shave margins and intraoperative radiography.^10^ For example, a study utilizing the National Cancer Database (NCDB) reported an overall re-excision rate of 16.1% after BCS across 1226 facilities, with substantial variability: the 10th percentile was 6.6% while the 90th percentile was 25%. The study identified the facility a patient was being treated at as the strongest determinant of re-excision rates.^12^ Similarly, an analysis of 291,065 Medicare claims found an overall re-excision rate of 17.2% after implementation of the 2014 ‘no tumor on ink’ guideline; however, individual surgeon re-excision rates varied widely from 0 to 91.7%.^11^ In attempt to account for the variability existing between different institutions and surgeons, we compared the re-excision rate in the current trial with a group of contemporary patients treated at our institution who also underwent BCS and WBRT, revealing a *p* value of 0.015. A comparison with the re-excision rate from the postoperative boost trial conducted several years ago was not performed as those patients were not contemporary.

We have also demonstrated that patients in the current trial had a significantly shorter interval from initial diagnostic biopsy to completion of radiotherapy in comparison with a contemporary cohort of patients treated at our institution with a standard postoperative boost, and with patients treated in a previous clinical trial at our institution using the same fractionation scheme but with a postoperative boost. The median treatment time in the current trial was about 2 weeks shorter than the median treatment time of both comparison groups. Decreased treatment time has many potential benefits, including being more convenient and less emotionally taxing for the patient, and allowing for the earlier initiation of systemic therapies, which could positively impact disease control outcomes. One major factor influencing this observed decrease in treatment time is that radiotherapy at our institution was generally able to be initiated in a shorter time frame than surgery was able to be scheduled—this was actually an appealing aspect for patients considering participation in this trial. A strength of this aspect of our study is the comparison with both a contemporary cohort and a previous clinical trial, as the timing of treatment may not be as strict in the non-clinical trial setting.

We acknowledge that a majority of these patients were low-risk patients in a relatively older age group (mean age 63 years) who did not necessarily ‘require’ any radiation. However, the majority of these patients were in excellent health with a long life expectancy, and at our institutions, these patients, although often offered observation as an option, choose radiation therapy to minimize the risk of a local recurrence over the course of their lifetime. In addition, in this low-risk patient population, while the shorter time to initiation of local-regional therapy and the shorter time to completion of local treatment may not be clinically or biologically a significant benefit, the shorter time to therapy was appreciated by the patient population.

Current evidence suggests that patients undergoing oncoplastic reduction mammoplasty (ORM) frequently have omission of a postoperative tumor bed boost, potentially compromising local control in patients who may benefit from the boost. This omission is often attributed to the difficulty in delineating the tumor bed after ORM.^9,17^ Among patients who receive radiotherapy following ORM, one study reported that 31% of patients had delayed initiation of radiotherapy, which was defined as more than 8 weeks between surgery and the start of radiotherapy.^18^ Thus, a preoperative boost could mitigate these challenges by reducing the need for postoperative tumor bed delineation and shortening overall treatment time for patients.

Of note, Dong et al.^19^ reported the results of a phase II study in which a preoperative radiation boost was delivered to 36 patients undergoing oncoplastic surgery for breast cancer treatment. Patients in this study received a preoperative boost of 10 Gy in one fraction with intensity modulated radiotherapy (IMRT) planning, followed by surgery, followed by WBRT of 26 Gy in 5 fractions. The study reported that compared with patients receiving a preoperative boost, control patients were more likely to report higher scores in chest physical well-being (*p* = 0.045) and attitude towards arm swelling (*p* = 0.01) per the results of the BREAST-Q questionnaire; however, the radiation treatment regimen received by the control group was unclear. Additionally, assuming an α/β of 4 for tumor control, the equivalent dose in 2 Gy fractions (EQD2) for their study’s boost was 23.33 Gy, while the EQD2 in our preoperative boost trial was 16.27 (13.32 Gy delivered over 4 fractions). Assuming an α/β of 2 for fibrosis, the EQD2 of the boost in the study by Dong et al. would be 30, while the EQD2 in our study was 17.5. The high EQD2 in this study may explain the lesser satisfaction with chest physical well-being and arm swelling in the treatment group.^19^

## Conclusion

Although the results of our study are encouraging, limitations include the single-institution setting and the relatively small sample size. Comparison of re-excisions rates and treatment time in a larger, randomized setting would provide more conclusive evidence, such as in a randomized cooperative trial currently being considered that would investigate the delivery of a preoperative radiation boost in patients undergoing ORM.
